# Tubulohelical membrane arrays: From the initial observation to the elucidation of nanophysical properties and cellular function

**DOI:** 10.1186/1757-5036-3-13

**Published:** 2010-06-28

**Authors:** Siegfried Reipert, Józefa Wesierska-Gadek, Sebastian Wienerroither

**Affiliations:** 1 Department of Biochemistry and Cell Biology, Max F. Perutz Laboratories, University of Vienna, Dr. Bohrgasse 9, A-1030 Vienna, Austria; 2 Cell Cycle Regulation Group, Institute of Cancer Research, University Clinic of Internal Medicine, Medical University of Vienna, Borschkegasse 8 a, A-1090 Vienna, Austria; 3 Department of Microbiology, Immunobiology and Genetics, Max F. Perutz Laboratories, University of Vienna, Dr. Bohrgasse 9, A-1030 Vienna, Austria

## Abstract

Lipids undergo self-assembly to form ordered nonlamellar, nanoperiodic arrays both in vitro and in vivo. While engineering of such membrane arrays for technical devices is envisaged, we know little about their cellular function. Do they represent building blocks of an inherent cellular nanotechnology? Prospects for answering this question could be improved if the nanophysical properties of the membrane arrays could be studied in the context of specific cellular functions. Therefore, we draw attention to exceptional complex membrane arrays found in the renal epithelial cell line PtK2 that could provide perfect conditions for both biophysical and cell functional studies. The so-called tubulohelical membrane arrays (TUHMAs) combine nanoperiodicity of lipid membranes with that of helix-like proteinaceous core structures. Strikingly, they show several characteristics of dynamic, microtubule-associated single organelles. Our initial data indicate that TUHMA formation occurs in the depth of the cytoplasm under participation of cytoplasmic nucleoporins. Once matured, they may fuse with the nuclear membrane in polarized positions, either perpendicularly or in parallel to the nucleus. As a starting point for the initiation of functional studies we found a connection between TUHMAs and primary cilia, indicated by immunolabeling patterns of detyrosynated tubulin and cytoplasmic nucleoporins. We discuss these observations in the context of the ciliary cycle and of the specific requirement of ciliated renal epithelial cells for oriented cell division. Finally, we raise the question of whether putative nanooptical properties of TUHMAs could serve for communicating orientation between dividing cells.

**MCS codes:** 92C37, 92C05, 92C50

## Findings

Cells have internalized self-assembly of lipid-phase dependent membranes as part of their life-organizing strategy. The diversity of lipid membrane structures has been highlighted by Tresset [1]. Among them, nanoperiodic 3D-arrays, so-called cubic phases, are of particular interest, since they inspire to a technical route to the *in vitro*-fabrication of 3D structures [2,3]. Whereas engineering of a man-made functional design is envisaged, it is unclear what the purpose of such membrane arrays in living cells might be. Do these arrays make use of physical properties resulting from their nanoarchitecture? Do they act as building blocks for cellular "technologies", yet to be conceived by humans? With next to no knowledge of the function of cellular nanoarrays, addressing such questions is difficult.

Here we want to raise the interest of biophysicists in a novel lipid membrane array since this could open prospects for studies of their self-assembly in relation to their cellular function. Initially, we observed this membrane array in the renal epithelial cell line PtK2, processed by state-of-the-art preparation techniques for electron microscopy (EM) [4]. Since it differs from any of the other highly ordered membrane structures reported previously, we named it the "tubulohelical membrane array" (TUHMA).

### The structural preservation of TUHMAs for transmission electron microscopy

Complex cellular lipid membrane structures and arrays, including those displaying nonlamellar aspects, are visible after conventional chemical fixation with cross-linking aldehydes followed by dehydration and subsequent embedding in epoxy resin. For viewing in the electron microscope, the resulting thin sections of the embedded samples are contrasted with heavy metals. The visibility of the lipid membranes in such preparations relies on the retention of lipids by post-fixation with osmium tetroxide. Accordingly, almost all observation of lipid membranes of higher order, such as organized smooth endoplasmic reticulum (OSER), tubuloreticular structures (TRS) and cubic membranes, originate from cells and tissues that are fixed with glutaraldehyde and post-fixed with osmium tetroxide [5-7]. While the preservation of these structures could possibly be improved by application of rapid fixation- and cryopreparation techniques, such state-of-the-art methods have not become standard yet in this field of research.

Similar to other membrane arrays, TUHMAs can also be observed in cells that are conventionally fixed with glutaraldehyde. For studying them in relation to other organelles, however, we preferred microwave (MW)-accelerated chemical fixation. While immersed in the fixative, the cell monolayers were subjected to MW exposure. Excessive warming-up of the samples was prevented by limitation of the number of MW pulses. The resulting structural improvements of membranous and cytoskeletal aspects of the cells are described elsewhere [8]. Notably, these results were achieved with lower concentrations of glutaraldehyde than used in conventional fixation.

To exclude the possibility that glutaraldehyde cross-linkage could have resulted in artifacts, we also processed cells without using this fixative. Cells were high-pressure frozen rapidly, followed by freeze substitution and low-temperature fixation with acetone as a coagulating solvent prior to embedding in epoxy resin [9]. TUHMAs were still observed in the absence of chemical cross-linkers, under conditions of cryopreservation [4].

### TUHMA structure: 3D-nanoperiodicity of both lipids and proteins, combined in an organelle-like entity

**Figure 1:**
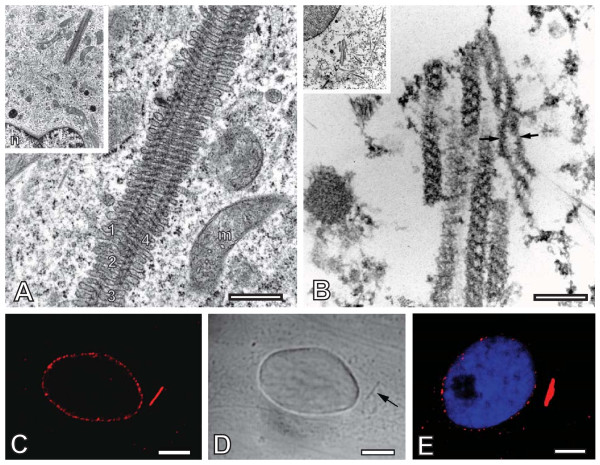
**The tubulohelical membrane array (TUHMA) visualized by transmission electron microscopy and light microscopy**. A) Processing by microwave-accelerated fixation with 0.5% glutaraldehyde in buffer solution, followed by treatment with OsO_4_, and embedding in epoxy resin for thin sectioning [8]. The organelle displays darkly contrasted tubules of uniform diameter of 80 nm, numbered 1-4. The tubules are incorporated in a regular stack of membranes and confined by helical bands that contrast in black. Note also a twisting pattern of the tubules within the plane of section and the size relation of the TUHMA in comparison with a mitochondrion, m. (courtesy of *Cell Biol Intern *[4]). B) Processing by microwave-accelerated fixation with 0.5% paraformaldehyde buffer solution and extraction with 0.1% Triton X-100, followed by treatment with OsO_4 _prior to embedding and thin sectioning. While the lipid membranes are dissolved, the proteinaceous core tubules remain intact exposing their helix-like aspects with great clarity. The core tubule on the right seems to detangle into separate threads (black arrows) giving the impression of a double helix bridged by delicate filaments. Bars in A and B, 500 nm. C) Confocal image of a cell immunolabeled with mAb 414. In the cytoplasm a Texas Red-fluorescent tubular structure is apparent. D) A tubular pattern in the DIC-contrast (arrow) co-localizes with the fluorescent tubular structure in C. Note that the cell in C, D was fixed and extracted with Triton X-100 in the same way as the EM sample in B. E) Confocal section of a cell indirectly immunolabeled with human autoimmune Abs against Nup62 and Texas Red-conjugated Abs, and counterstained with Hoechst 33258. Note that both the mAb 414 and the autoimmune Abs label NPCs at the nuclear envelope. Bars in C-E, 5 μm.

TUHMAs are nonlammellar, nanoperiodic lipid membrane arrays organized around tubular, proteinaceous electron-dense cores. The interface between the core tubules (80 nm in diameter) and the lipid membranes displays characteristic helix-like threads which appear dark in contrast. Depending on the cell status, up to 8 core tubules provide the basis for an intermingled, nonlamellar membrane scaffold of an overall length of 3-5 μm (Fig. 1A). The nanoarchitecture of TUHMAs lacks the high-degree of symmetry characteristic of cubic membranes [5]. This observation might be of interest to biophysicists who would prefer asymmetric structures as a prerequisite for broad application in photonics [2]. Notably, the asymmetric nanoarchitecture of TUHMAs results from a combination of both lipid-and protein nanoperiodicity. Treatment with the detergent Triton-X100 exposes the helix-like threads lining the core tubules of TUHMAs (Fig. 1B).

### Nucleoporins: Could they serve as templates for lipid self-assembly in TUHMA formation?

The monoclonal antibody 414 raised against a set of nucleoporins is a proven marker of TUHMAs in the light microscope [4]. Even after dissolution of their membranes with Triton X-100, the immunofluorescence signal of TUHMAs remains strong (Fig. 1C), co-localizing perfectly with tubular entities resembling TUHMAs in the DIC mode of the light microscope (Fig. 1D). Therefore, we expect the epitopes recognized by mAb 414 to be associated with the core tubules of TUHMAs. The diameter of these tubules is similar to the diameter of nuclear pore complexes (NPCs), making the participation of nucleoporins in the architecture of TUHMAs conceivable. To substantiate the claim that nucleoporins are involved, we labelled TUHMAs with human autoimmune antibodies against Nup62. Nup62 is located at the inner pore channel of the NPCs [10,11], and it is among the nucleoporins that are recognized by mAb 414 [12]. The intense labelling with autoimmune antibodies indicates that Nup62 could indeed be a major component of TUHMAs (Fig. 1E).

Labeling of TUHMAs for nucleoporins comes less as a surprise since they are transiently associated with the nucleus [4]. Therefore, one may anticipate that helix-like, nucleoporin-containing structures extrude from the nuclear membrane, in this way creating templates for the formation of the lipid membrane array. This would be compatible, at least in part, with the postulation by Wheatley [13] that helical pore complexes are released from cell nuclei into the cytoplasm. However, we found no indications at all of outgrowth of core tubules from the nucleus. Therefore, we conclude that the assembly of TUHMAs takes place within the cytoplasm. The questions remaining to be answered are how nucleoporins could contribute to such a process without any apparent link to the nucleus, and how they establish an interaction with lipids.

### A hypothesis for TUHMA formation: Pleomorphic membrane changes under participation of cytoplasmic nucleoporins

**Figure 2:**
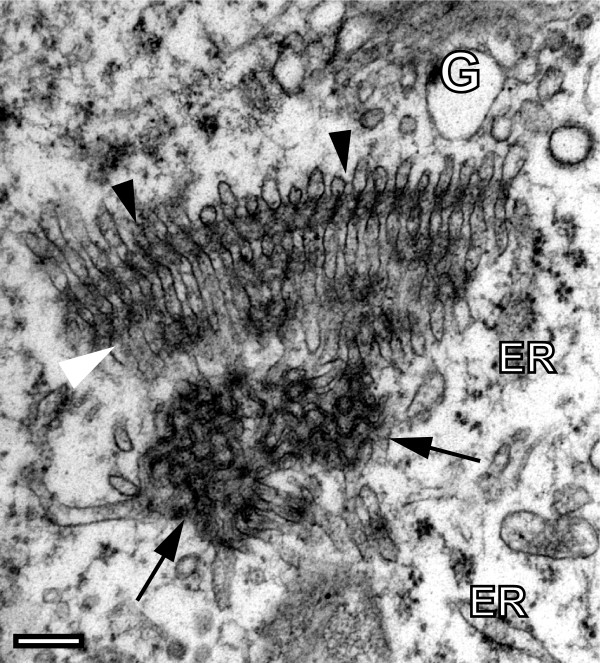
**Transitional stage of lipid assembly at the periphery of a TUHMA**. Irregular tubulo-reticular structures (TRS) at the periphery of a TUHMA which contains half-opened darkly contrasted loops that resemble building elements of cubic membranes (arrows). Between the core tubule of the TUHMA (black arrowheads) and the TRS exists a transitional zone (white arrowhead) displaying interleaves between putative elements of emerging or disappearing core elements. Note also rough endoplasmic reticulum (ER) and extensions of the nearby Golgi complex (G). Bar, 200 nm.

We suggest that TUHMAs are the result of pleomorphic changes of reticulated membrane domains [6] under interaction with cytoplasmic nucleoporins in membrane-bound and, perhaps, soluble form [14,15] and the Golgi complex. The observation of annulate lamellae (AL), endowed with nucleoporin-containing pore complexes in association with TUHMAs supports this idea. Moreover, we found unusual nonlamellar membrane structures, called tubuloreticular structures (TRS), linked to TUHMAs that have not yet been analysed for nucleoporin content [5]. Some of them display aspects which resemble cubic membranes (Fig. 2). We regard them as transitional stages of the lipid assembly at the periphery of the lipid membrane array. The TRS seem to bridge the space between the TUHMA and the Golgi complex [4] indicating an involvement of the latter in TUHMA formation. Interestingly, the Golgi complex can also be physically linked to nucleoporin-containing AL [16].

### TUHMA dynamics: Prospects for studies of cell-cycle coupled self-assembly

TUHMAs are single organelle-like entities observed in just 5-10% of the cells of an asynchronously grown cell population. They preferentially occupy a polarized position either oriented perpendicularly or in parallel to the cell nucleus. Furthermore, their varying position relative to other organelles indicates that they are capable of motility. Since TUHMAs are linked to microtubules (MTs), such dynamics might be driven by motor proteins associated with them [4].

Studies of individual sub-clones of PtK2 cells indicate that TUHMA dynamics is coupled with the cell cycle. It is notable that even after two cell divisions THUMAs are synchronically arranged with respect to their distance to the nuclei and their orientation. Therefore, we are looking forward to the dissection of stages of TUHMA biogenesis and dynamics in synchronized cells. Given the low number of "mature" TUHMAs in asynchronously grown cell populations, we expect TUHMAs to be enriched in synchronized S-phase and prophase cells. Once bound to the nucleus at prophase the breakdown of TUHMAs could coincide with that of the nuclear envelope during mitosis.

### TUHMA function: Preliminary observations as a starting point for systematic studies of the ciliary cycle

Double labelling of TUHMAs and α-tubulin in proliferating PtK2 cells indicates that, under conditions not yet specified, centrosomes, TUHMAs, and primary cilia display proximal positioning to one another. Are these observations coincidental or do they indicate a coupling of the TUHMA dynamics to the ciliary cycle (for review: [17,18])?

**Figure 3:**
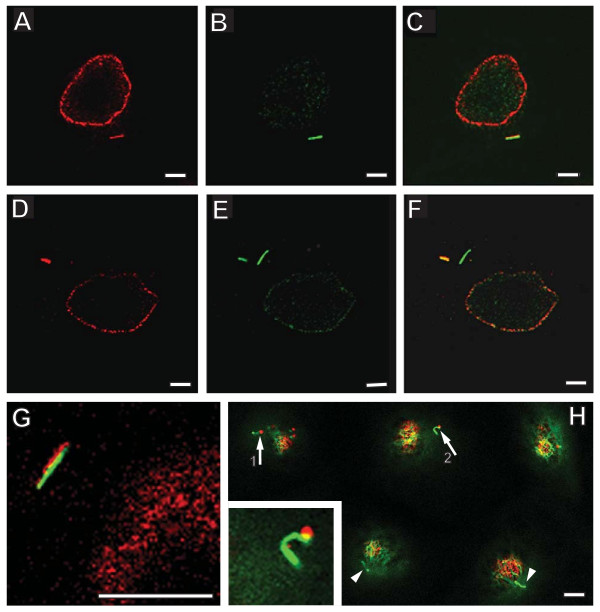
**The connection between TUHMAs and primary cilia indicated by double immunolabeling with antibodies against tubulins and nucleoporins**. A) A TUHMA labelled with mAb 414 (red) and B) tubule-forming detyr-T (green) both located in the same confocal section close to the substratum of cell growth. C) overlay of A and B indicating the co-localization of both fluorescence signals. D) TUHMA labelled with mAb 414 (red; arrow) and E) detyr-T (green) forming two tubules positioned orthogonally to each other both located in the same confocal section close to the substratum of cell growth. F) Overlay of D and E indicating that just one of the detyr-T containing tubules co-localizes with the TUHMA in D. G) Co-localization of a tubular structure labelled with mAb 414 and detyr-T in an upper confocal section perhaps harbouring the axoneme. F) Cilia in an upper confocal section: Two short cilia (arrows), numbered 1 and 2, labelled with antibodies against α-tubulin (green) are associated with red fluorescent dots resulting from labelling with mAb 414 raised against nucleoporins. The insert displays cilium 2 in more detail. In contrast to cilia 1 and 2, two longer cilia in neighbouring cells (arrowheads) are free of labelling with mAb 414. Bars, 5 μm.

To ensure specificity in labelling of cilia we applied antibodies against detyrosinated tubulin (deTyr-T) [19]. Strikingly, we found diverse stages of co-localization between deTyr-T and TUHMAs in confocal sections close to the substratum of cell growth (Fig. 3 A-F). Moreover, we became aware of occasional co-localization of deTyr-T and mAb 414 at upper section planes containing the axoneme of cilia. Are these indications of a MT-guided translocation of nucleoporins between TUHMAs and primary cilia? This indeed could be the case, since we found short cilia with spot-like labelling patterns of mAb 414 among ciliated cells (Fig. 3 H). In contrast, mature, elongated cilia in quiescent cells resulting from postconfluent cell growth were not labelled with mAb 414. Notably, TUHMAs were almost absent in postconfluent cell populations.

Taken together, our preliminary data indicate a relationship between primary cilia and TUHMAs that awaits systematic interdisciplinary studies. They contain two surprising aspects: i) the cooperation between the membrane array and tubulin enriched in deTyr-T usually seen in conjunction with 'long lived' stable MTs [20], and ii) the translocation of proteins recognized by antibodies against nucleoporins. At first glance, nucleoporins playing a role in the ciliary cycle appears to be far-fetched. However, nucleoporins might fulfil structural requirements made necessary because of their common ancestry with intraflagellar transport protein complexes [21]. In spite of this, a connection between nucleoporins, TUHMAs and cilia is unlikely to be directly related to ciliary function as a sensory organ. This can be concluded from the lack of nucleoporin labeling in mature primary cilia, the almost complete absence of TUHMAs in postconfluently grown cells with well-developed cilia, and from the fact that TUHMAs have not been reported for ciliated tissues so far. Since tissues are represented dominantly by cells in a quiescent state, the search for putative functions of TUHMAs has to be focused on studies of the cell and ciliary cycles in cell culture models.

For the reasons stated above we conclude that TUHMAs do not exist at the beginning of the ciliognesis in the early G1 stage of the cell cycle [17]. Could they play a role instead in preparing ciliated cells for S-phase? Preparation for the synthesis phase is marked by significant structural alteration including the resorbtion of cilia and the beginning of the reorganization of cytoplasmic MT complex in preparation for mitosis [22,23]. Could such processes profit from the existence of an anisotropic nanoperiodic membrane array?

A closer look at renal epithelium, the tissue origin of the PtK2 cells in which TUHMAs were found, indicates particular requirements for cell division and polarity *in vivo*. To form elongated renal tubes, the epithelial cells have to undergo oriented cell division based on mitotic spindle alignment, and they have to ensure a planar cell polarity [24]. Otherwise, the renal tubes will enlarge and form cysts, with life-threatening consequences. The principles according to which cells generate and maintain such ordered structures are not known. It has also been suggested there may be sophisticated, GPS-like, communication techniques between the renal epithelial cells; however hypotheses as to how such a communication could be realized are vague at best [24,25]. Essentially, they must rely on organelles with the ability to sense directed signals, such as primary cilia [26]. Fischer and Pontoglio (2009) speculated that sensing of the urine flow by primary cilia could provide a cue for building up of anisotrophy within the cytoskeleton, which might affect cell division [24]. How this really happens remains unclear. Therefore, we wonder whether TUHMAs might help to explain oriented cell growth and planar cell polarity.

In terms of cell-and molecular biology, future studies of TUHMAs will include their isolation for proteomic analysis, study of their biogenesis, reconstruction of their dynamics in relation to the cell and ciliary cycle, and the identification of cell physiological and pathological parameters related to their self-assembly. The question of whether or not these lipid nanoarrays assemble for functional purpose, however, requires the input of biophysicists. In particular, we would like to encourage them to evaluate and study putative nanooptical properties of TUHMAs. In doing this they may find electromagnetic absorption at wavelengths relevant to a novel way of cellular communication and/or ways for visualizing TUHMAs by live-cell imaging.
